# Flexible Temperature Sensors

**DOI:** 10.3389/fchem.2021.539678

**Published:** 2021-09-22

**Authors:** Ruping Liu, Liang He, Meijuan Cao, Zhicheng Sun, Ruiqi Zhu, Ye Li

**Affiliations:** ^1^ Beijing Institute of Graphic Communication, Beijing, China; ^2^ State Key Laboratory of Advanced Technology for Materials Synthesis and Processing, Wuhan University of Technology, Wuhan, China

**Keywords:** sensor, nanomaterials, fabrication, energy storage, temperature

## Abstract

Temperature reflects the balance between production and dissipate of heat. Flexible temperature sensors are primary sensors used for temperature monitoring. To obtain real-time and accurate information of temperature, different flexible temperature sensors are developed according to the principle of flexible resistance temperature detector (FRTC), flexible thermocouple, flexible thermistor and flexible thermochromic, showing great potential in energy conversion and storage. In order to obtain high integration and multifunction, various flexible temperature sensors are studied and optimized, including active-matrix flexible temperature sensor, self-powered flexible temperature sensor, self-healing flexible temperature sensor and self-cleaning flexible temperature sensor. This review focuses on the structure, material, fabrication and performance of flexible temperature sensors. Also, some typical applications of flexible temperature sensors are discussed and summarized.

## Introduction

To the best of our knowledge, sensor is the key of various induction equipments, and a critical issue with great potential. In the past decade, a great progress of sensors in many fields is achieved. Sensors with induction feature are playing increasing important roles in various fields, such as medical monitoring, industrial production, wearable equipment, internet of things (IoT), etc (Cheng et al., 2020; Kai et al., 2020; Kun et al., 2020; Shao et al., 2020). One important kind of sensors in induction equipment is the flexible temperature sensor. Flexible sensor is a kind of sensor made of flexible material, which has high flexibility, high ductility, even free bending or folding (Abdelmoughni et al., 2020). It can be arranged arbitrarily, and can detect complex units easily. New types of flexible temperature equipments, textiles, aerospace, environmental medical care, electronics, electricians, sports sensors are widely applied in electronic skin and monitor, etc (Zamri et al., 2015; Jea Sang et al., 2020; Jian et al., 2020; Su et al., 2020; Ye et al., 2020).

A complicated interplay of various regions of flexible temperature sensors is required for flexibility of the most basic feature. In addition, the robots with flexible temperature sensors have increased control over their action. In this review, we summarized the structure, material, fabrication and performance of flexible temperature sensors. We also elaborated the most widely accepted theory concerning the flexible temperature sensors and the evidence supporting this theory. Finally, we reviewed the applications of flexible temperature sensors in various fields, especially in power system, industrial production and medical device.

## Traditional Flexible Temperature Sensors

Structure, material, fabrication and performance are important factors of flexible temperature sensors. Development of flexible temperature sensors with digitalization and intelligence is still a great challenge. Previous studies found that the structure, material and fabrication process have great influences on the performance of sensors (J Mittemeijer, 2011; Nosbi et al., 2010; Chen et al., 2017a). It is noted that this trend is consistent with the design of flexible temperature sensors. Various flexible temperature sensors are developed according to the principles, such as flexible resistance temperature detector (FRTC), flexible thermocouple, flexible thermistor, flexible thermochromic (Ying et al., 2011; Zhang et al., 2017). This section will introduce several typical flexible temperature sensors.

### Flexible Resistance Temperature Detector

FRTC is the most common flexible temperature sensor. In particular, FRTC converts the applied temperature into electrical signal, which has been widely explored. Monitoring health conditions of the human’s body *via* detecting the subtle temperature variation related with human’s activities is possible, such as the body’s temperature. High sensitivity, high flexibility, and excellent reliability are required for FRTC in practical applications (Chen et al., 2017a; Zhang et al., 2017). To achieve high-performance FRTC, considerable efforts have been made in optimization of the materials and device configurations. First, various active materials such as graphene, carbon black (CB), carbon fiber, carbon nanotube (CNT) and multi-walled CNT (MWCNT) (Liu et al., 2012; Guo et al., 2014; Tian et al., 2015; Wang et al., 2017; Wu et al., 2019) have been introduced into FRTC as the conductive fillers due to their high conductivity, low cost, and high stability (Kun et al., 2020; Abdelmoughni et al., 2020; Jea Sang et al., 2020; Su et al., 2020; Jian et al., 2020; Ye et al., 2020; Zamri et al., 2015; J Mittemeijer, 2011; Nosbi et al., 2010; Chen et al., 2017a; Zhang et al., 2017a; Ying et al., 2011; Wang et al., 2017). Second, for obtaining highly flexible and stretchable devices, polymers including polydimethylsiloxane (PDMS) (Shih et al., 2010; Sibinski et al., 2010; Zhao et al., 2018a), silicon rubber, poly (vinylidene fluoride) (PVDF), polymethyl methacrylate (PMMA) and poly (3,4-ethylenedioxythiophene-poly (styrenesulfonate) (PEDOT: PSS) (Nakata and Arie, 2017; Huang et al., 2018; Shen et al., 2018; Chen et al., 2018; Bang et al., 2019) have been widely investigated in FRTC (Shih et al., 2010; Sibinski et al., 2010; Liu et al., 2012; Guo et al., 2014; Tian et al., 2015; Nakata and Arie, 2017; Wang et al., 2017; Zhao et al., 2018a; Huang et al., 2018; Shen et al., 2018; Chen et al., 2018; Bang et al., 2019; Wu et al., 2019). It is demonstrated that preparation of the polymer merits and the sensing layer of conductive materials is a highly promising way for fabrication of high-performance FRTC. Nano/micro porous structures are applied to obtain sensors with increased sensitivity and improved response speed (Nakata and Arie, 2017; Shen et al., 2018).

The temperature coefficient of resistance (TCR) of most metals is between 0.01 and 0.1°C^−1^, and similarly, other conductive materials, e.g., the CNTs incorporated with PEDOT: PSS (Nakata and Arie, 2017; Shen et al., 2018), also exhibited a comparable sensitivity. Applying the percolation effect is a possible strategy for obtaining enhanced sensitivity of temperature sensor (Shen et al., 2018), significantly decreasing resistance of the FRTC by several orders through filling a conductive material into an insulating polymer matrix, e.g., PDMS and silicon rubber (Sibinski et al., 2010). Although percolation-type FRTC typically offers an ultrahigh ΔR/R value, as described in Table 1, this resistance change typically occurs at a narrow range of temperature, limiting their applications in wide-range temperature sensing. Different from the narrow operating temperature, the FRTC focuses on a broader sensing range of 20–100°C. Piezoelectric polymer matrix (such as PVDF) and conductive polymer matrix (such as PEDOT: PSS) can also be applied in temperature sensing.

**TABLE 1:** Comparison of flexible resistance temperature detectors.

Device	Material	TCR (°C^−1^)	Temperature change (°C)	PTC or NTC	References
5 × 5	Silicon rubber-carbon fiber	0.1823	25–70	PTC	Zhao et al. (2018a)
1 × 1	Silicon rubber-CB, CNTs	0.00572	20–80	PTC	Wang et al. (2017)
1 × 1	Silicon rubber-CB	10^–4^	0–50	PTC	Liu et al. (2012)
3 × 3	Silicon rubber-carbon fiber	0.0394	20–80	PTC	Guo et al. (2014)
12 × 12	Silicon rubber-CB, graphene	0.0327	25–90	PTC	Tian et al. (2015)
1 × 1	PDMS-the flake graphite, CNT	0.028	35–85	PTC	Wu et al. (2019)
1 × 1	PDMS-porous carbon	0.11	23–50	PTC	Zhao et al. (2018a)
4 × 4	PDMS-graphite	0.0055	30–110	PTC	Shih et al. (2010)
1 × 1	PMMA-MWCNTs	0.0013	30–42	NTC	Sibinski et al. (2010)
10 × 10	PVDF-MWCNTs, PEN	0.081	25–100	NTC	Chen et al. (2018)
1 × 1	PVDF-graphite, PEO	0.1	25–42	PTC & NTC	Huang et al. (2018)
1 × 1	PEDOT: PSS-CNT	0.0078	20–60	NTC	Bang et al. (2019)
1 × 1	PEDOT: PESS-CNT	31	10–50	NTC	Nakata et al. (2017)

#### Insulating Polymer Matrix

For research work about flexible temperature sensors, it is found that the conductive composites are often applied as the sensing materials of FRTC. In the past several years, some conductive composites containing dispersed conducting carbon nanomaterials in an insulating polymer matrix are investigated for resistance temperature detectors. The conducting carbon nanomaterials include carbon fiber, graphene, porous carbon, silver nanoparticle (NP) and CNT, etc., and the polymer matrices include silicon rubber and PDMS. Resistance temperature detectors are fabricated by bonding the interdigital electrodes and conductive composites with conductive silver glue (Liang et al., 2015). The electrical resistivity of these composites is critically dependent on the volume fraction of conducting filler, well explained by percolation theory. With the increase of temperature, the conductive network chains of conductive composites are destroyed. Additionally, the volume expansion of polymer matrix leads to the decrease of the volume fraction of conducting carbon nanomaterials indirectly, resulting in the increase of bulk electrical resistivity of conductive composites. It shows a characteristic of positive temperature-resistance coefficient (PTC). A new conductive composite is proposed and enables production through screen printing. The new conductive composite is based on conductive material-polymer paste, consisting of PMMA employed as the binder. The PMMA was dissolved in organic solvents at elevated temperature, until a homogenous consistence was achieved. Then MWCNTs were added and mixed with a three-roller mill. The agglomerate sizes of below 10 μm are obtained *via* rolling. High temperature coefficients are utilized to characterize these temperature sensors, reaching 0.0013°C^−1^ in 30–42°C. It shows a characteristic of negative temperature-resistance coefficient (NTC) (Wu et al., 2019).

**FIGURE 1:**
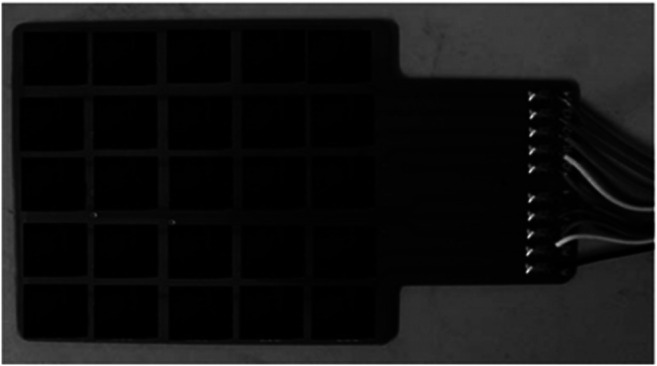
Flexible temperature sensor array (Huang et al., 2012).

Huang and co-workers Huang et al. (2012) proposed a FRTC array by sticking the sensing materials of the conductive composites formed by the silicone rubber and carbon fiber into the interdigital electrodes with conductive silver glue. Figure 1 shows a schematic of this flexible temperature sensor with electrode-substrate-sensing material sandwiched structure. The study results showed that the relationship of resistance of the flexible temperature sensor and distance between sensor and temperature source is linear, and the repeatability of the experimental results is good. Moreover, the resistance of the flexible temperature sensor varies linearly with the ambient temperature between 25 and 70°C. The researchers also discussed the effects of conductive composites with different carbon fiber contents on flexible temperature sensor.

**FIGURE 2:**
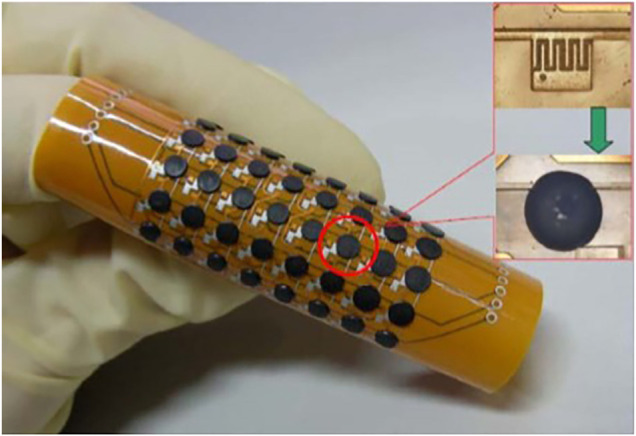
Flexible temperature sensor array (Shih et al., 2010).

Tsao group (Shih et al., 2010) presented a new method for fabricating passive-matrix FRTC array. They dispersed a graphite-PDMS composite on interdigitated copper electrodes patterned on flexible polyimide films. The flexible temperature sensor array with electrode-substrate-sensing material sandwiched structure shown in Figure 2 has 64 sensing cells in an area of 16 cm^2^. Their investigation presented that graphite powder provided the composite high temperature sensitivity. In composites with different graphite volume fractions, they observed that the composite with 15% graphite powder is suitable for on/off devices while the one with 20% graphite powder provides sufficient dynamic range for continuously sensing the change of temperature.

#### PVDF Matrix

Recently some conductive composites containing dispersed conducting carbon nanomaterials in a piezoelectric polymer matrix have been studied for resistance temperature detectors. The conducting carbon nanomaterials are carbon fiber, graphene, graphene oxide, porous carbon, silver NP and CNT, etc., and the polymer matrices are PVDF (Huang et al., 2018; Bang et al., 2019). Resistance temperature detectors are fabricated by coating the conductive composites on interdigital electrodes using spinning and printing. Another approach is based on nano conductive material-polymer paste, consisting of polyethylene modified polystyrene and rubber as a binder material. These components were dissolved in organic solvents at elevated temperature, until a homogenous consistence is obtained. Afterward, nano conductive material as the filler was added and mixed in a three-roller mill. Rolling is performed until agglomerate sizes of below 10 μm are obtained. Several series of flexible temperature sensors are produced and tested. They have a characteristic of NTC. The functionality of polymer composites is improved through CNTs by enhancing their strength and thermal and electrical conductivities. The composites with CNTs can revolutionize structural materials’ design and production in construction elements. Potential applications in electronic circuits fabricated by printing techniques are smart clothing and flexible electronics including functional elements (e.g., printed transistors) or biochemical sensors. In previously conducted experiments related to CNT layers, a high resistance dependence on temperature is indicated, which allowed for experimenting in the textronic thermal sensory field.

Huang group (Huang et al., 2018) presented a FRTC consisting of graphite-filled polyethylene oxide (PEO) and PVDF sensing layer, silicon rubber substrate and PDMS covering layer, exhibiting a high accuracy of 0.1°C and perfect repeatability nearly 2,000 times in the sensing temperature range of 25–42°C. The FRTC was fabricated by the following procedure: first, fabricating sensing layer by dissolving the PEO in the deionized (DI) water using a magnetic stirrer for 1 h, then adding graphite powder to PEO/DI water solution followed by sonication for 1 h and magnetic stirring for 1 h. After that, PVDF and *N,N*-Dimethylformamide (DMF) were introduced and mixed for 3 h under heat treatment. The PEO/PVDF/graphite solution was dropped on the polyimide (PI) flexible substrate and coated uniformly using spin-coating. After drying the solution, the sensing layer on silicon rubber was removed and the silicon rubber was covered with PDMS. As electrodes, copper wires were bonded to the ends of FRTC using silver paste.

#### PEDOT: PSS Matrix

Recently some conductive composites containing dispersed conducting carbon nanomaterials in a conductive polymer matrix are studied for FRTCs. The conducting carbon nanomaterials are carbon fiber, graphene, graphene oxide, porous carbon, silver NP and CNTs, etc., and the typical polymer matrix is PEDOT: PSS (Kanao et al., 2015; Shen et al., 2018). Kanao group (Kanao et al., 2015) demonstrated a FRTC based on CNT ink and PEDOT: PSS solution. For the FRTC, the mixed ink consisting of CNT ink and PEDOT: PSS solution was printed on polyester (PET) substrate through the mixed ink over the polyester shadow mask after string and drying at 70°C for 60 min in air ambient. The maximum sensitivity of FRTC of ∼0.78%°C^−1^ at a weight percent ratio (3:1) of mixture is achieved. It showed a NTC characteristic.

### Thermistor

The resistance changes could be measured by flexible thermistors with high repeatability and accuracy, and can be easily integrated on one platform. Flexible thermistors are belonged to flexible temperature sensors based on metal film, semiconductor film and alloy film. The flexible thermistors on flexible substrates are fabricated by microelectromechanical system (MEMS) technology, flexible technology, printing technology and coating technology. Metal solder blocks are thought to act as the electrodes of the sensors for connecting conductive and transmission signals.

Flexible thermistors with thermal resistance films are fabricated on flexible PI, PET, or PDMS substrate, in which the thermal resistance films include platinum film, copper film, gold film, silver film, reduced graphene oxide (rGO) film, graphene film, graphene oxide film, silver nanowire (Ag NW) film, vanadium dioxide (VO_2_) film, CNT film, pentacene/silver NPs film, and silver nanocrystal film, etc (Xiao et al., 2005b; Jeong et al., 2010; Yokota et al., 2015a; Kanao et al., 2015; Guo et al., 2015; Kim et al., 2016; Zhao et al., 2018b; Trung et al., 2018; Chu et al., 2018; Zhu et al., 2018; Bang et al., 2019; Cui et al., 2019; Li et al., 2019). The PI, PET, PDMS and polyethylene naphthalate (PEN) substrates offer an excellent thermal insulation. The resistance of thermal resistance film changes with the temperature increasing. As Table 2 described, there is a comparison between different flexible thermistors.

**TABLE 2:** Comparison of different flexible thermistors.

Material	TCR (°C^−1^)	Temperature (°C)	PTC or NTC	References
Ag NWs	0.00294	25–60	PTC	Cui et al. (2019)
Ag nanocrystal	0.5	30–50	PTC	Bang et al. (2019)
rGO	0.0195	30–80	NTC	Zhao et al. (2018b)
rGO fiber	0.8	30–45	NTC	Trung et al. (2018)
Ag NWs	0.00286	30–80	NTC	Li et al. (2019)
Pt	0.0023	25–200	PTC	Xiao et al. (2015)
Au	1.4	25–92	PTC	Chu et al. (2018)
GNPs	0.0371	20–80	PTC	Le et al. (2017)
MWCNTs	0.034	20–80	PTC	Le et al. (2017)
Cu	0.00273	20–90	PTC	He et al. (2018)
Ag	0.002	−20–175	PTC	He et al. (2018)

(He et al., 2018) presented a copper flexible thermistor and a platinum film flexible temperature sensor based on serpentine structure (Figure 3). The experimental results demonstrated that the sensitivity of the copper film flexible temperature sensor is about 0.0027°C^−1^ while the sensitivity of the one with serpentine is about 0.00136°C^−1^. The study indicated that the sensitivity of the platinum film flexible temperature sensor is about 0.00273°C^−1^ while the sensitivity of the one with serpentine is about 0.00235°C^−1^. Ting group (Ting, 2015) investigated two Ag film flexible thermistors based on different structures, as shown in Figure 3. The obtained results indicated that the sensitivity is about 0.002°C^−1^, and the largest hysteresis is smaller than 1%. In addition, the response time is several 10 seconds (Ting, 2015).

**FIGURE 3:**
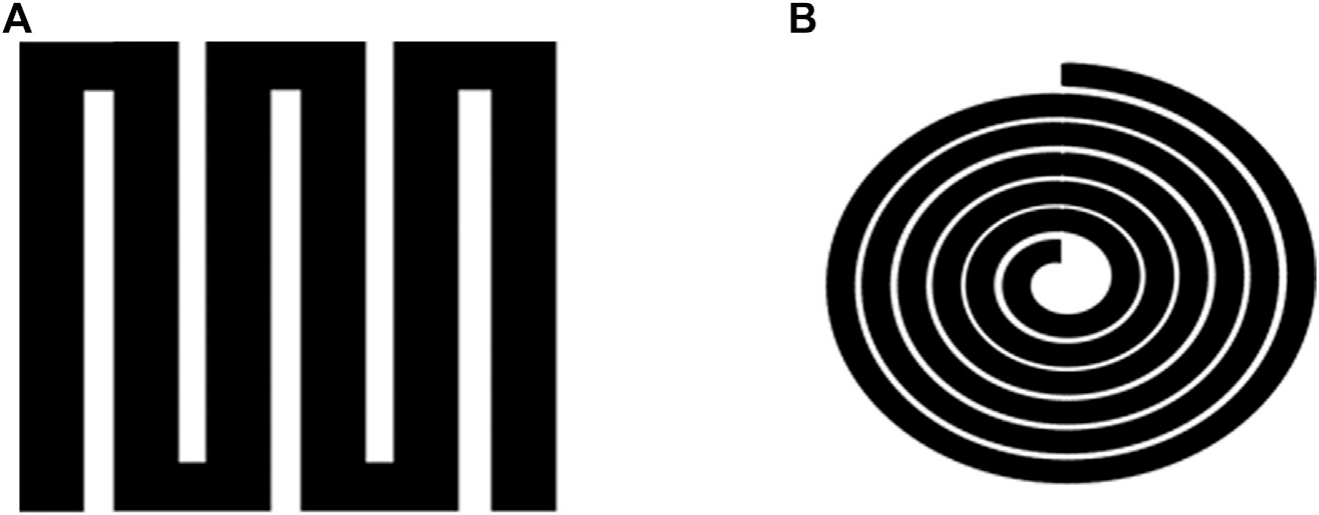
Structure diagram of thermistor. **(A)** Spiral structure and **(B)** serpentine structure.

### Thermocouple

Flexible thermocouples are belonged to flexible temperature sensors based on alloy film. The flexible thermocouples on flexible substrates are fabricated based on MEMS technology, printing technology or coating technology. Metal solder blocks are thought to act as the electrodes of the sensors for connecting conductive and transmission signals functions. Flexible thermocouples with thermocouple alloy films are fabricated on flexible PI or PDMS substrate, where the thermocouple alloy films are nickel-aluminum-silicon-manganese alloy film, nickel-aluminum alloy film, p-Sb_2_Te_3_ film, n-Bi_3_Te_3_ film, Bi-Te film and Sb-Te film, etc (Pan et al., 2018; Huynh and Haick, 2018; Su and Shen, 2019). The electrodes of thermocouple temperature sensors are usually prepared from metal films. When the alloy films of two different components are combined into a circuit and the temperature of the two junction points is different, a thermoelectric potential will be generated in the circuit (Trung et al., 2018). By measuring temperature-dependent voltage at the junction of two distinct alloy films, the flexible thermocouple can sense the temperature (Bell, 2008; Martin et al., 2010; Su and Shen, 2019).

X. Pan and co-workers (Pan et al., 2018) presented a flexible thermocouple to monitor the *in-situ* temperature of ion battery. In this flexible thermocouple, the PI is served as flexible substrate, the nickel-aluminum-silicon-manganese alloy film and nickel-aluminum alloy film are served as sensing materials, and the copper film is utilized as electrode. The experimental results showed that in different charge-discharge cycles of batteries, the measurement results of this film thermocouple temperature sensor are consistent with those of ARC instrument.

Huynh group (Huynh et al., 2018) reported a flexible temperature thermocouple consisting of p-Sb_2_Te_3_ and n-Bi_3_Te_3_ for health monitoring. As the most widely used thermoelectric materials, p-Sb_2_Te_3_ and n-Bi_3_Te_3_ have high thermoelectric efficiency at room temperature. Based on proof-of-concept prototype, flexible thermocouples of p-Sb_2_Te_3_ and n-Bi_2_Te_3_ arrays are sputtered on polyimide substrate.

### Thermochromic

Thermochromic materials have extensive potential applications in temperature sensors and have received increasing attention. Thermochromic materials are important functional and smart materials. When heated or cooled, the thermochromic materials will possess a thermal memory function, then the color of those will emerge pronounced changes. Comparing the color of thermochromic materials with that of standard color, it is easy and quick to know the surface temperature of measured objects (Li et al., 2019; Zhang et al., 2017; Geng et al., 2018; He et al., 2019).

He’s group (He et al., 2019) performed experimental investigations on developing a dressing and wearable flexible temperature sensor by dispersing thermochromic materials into the polyvinyl alcohol and water-soluble polyurethane composites. The prepared thermochromic materials are TC-M/NPCMs by chemical integration of trimesoyl chloride (TMC) and nanoencapsulated phase change materials (NPCMs), exhibiting excellent temperature indicator performance. The temperature on different positions of body surface is obtained by attaching the flexible temperature sensor to different positions of body surface and comparing its color with that of standard color (He et al., 2019).

### Polymer

Flexible temperature sensors have been investigated with several sensing materials such as polymer, graphene and CNT, etc. Polymers are employed to fabricate mechanically flexible temperature sensors, in which the polymers are acrylate copolymers, polyvinyl alcohol, etc (Das and Prusty, 2012; Honda et al., 2014; Borghetti et al., 2016; Yokota et al., 2015b). In particular, polymers can be easily synthesized by electrochemical polymerization, e.g., the potentiodynamic method. Cost effectiveness and uniform morphology are the distinctive advantages of electrochemical polymerization. The performance of polymers can be regulated through chemical treatment and doping. The polymers with positive temperature coefficient are utilized for fabricating mechanically flexible temperature sensors that have orders-of-magnitude changes in resistivity over only a few degrees. The need for per-pixel amplification circuitry can be eliminated by extraordinarily large changes in resistivity, as the sensor’s output signal can be directly multiplexed and fed to external recording instrument, ultimately decreasing the manufacturing cost and complexity of device.

Kim group (Kim et al., 2019) demonstrated a new type of flexible temperature sensor consisting of polyvinyl alcohol (PVA) function layer, aluminum oxide (Al_2_O_3_) encapsulating layer, flexible PEN substrate and sliver patterning electrodes. The fabricated flexible temperature sensor is based on conductive and uniform interdigital sliver patterning electrodes deposited on a flexible PEN substrate by printing technology with reverse offset. The PVA function layer is employed as temperature sensing material deposited by electrohydronamic atomization. The Al_2_O_3_ film is used as encapsulating layer deposited by spatial atmospheric atomic layer deposition (SAALD). The heating treatment on flexible temperature sensors was performed at 20–90°C in an inert environment with the help of dehumidifier inside the sealed chamber. Because of the PVA with negative temperature coefficient, its resistance decreases with the increase of temperature. They also discussed the different performances of the flexible temperature sensor encapsulated and non-encapsulated with Al_2_O_3_ film.

## New Type of Flexible Temperature Sensor

Flexible temperature sensors with multi-function and high integration have received more and more attentions. According to different functions, flexible temperature sensors can be divided into active-matrix flexible temperature sensor, self-powered flexible temperature sensor, self-healing flexible temperature sensor and self-cleaning flexible temperature sensor. Compared with ordinary flexible temperature sensors, functional flexible temperature sensors introduce new materials, new structures and new technologies, which enable the functional flexible temperature sensors not only to detect temperature, but also have other functions, such as self-power supply, self-healing, self-cleaning, etc (Mallory et al., 2013a; Mallory et al., 2013b).

Flexible temperature sensors play a critical role in early diagnosis *via* continuous monitoring of complicated conditions in health and disease. The stretchable, active-matrix, self-powered, self-healing and self-cleaning sensing systems are thus revolutionizing the sensors. The linkage of these technologies and advanced materials is particularly specified (Rogers et al., 2010; Yamamoto et al., 2017). Some weak and strong points in the development of flexible temperature sensor are clearly summarized and highlighted. Some aspects about further improvement of flexible temperature sensor are also discussed.

### Highly Accurate Flexible Temperature Sensors

The goal of accurate measurement of temperature is to reduce the detection error, which can more accurately detect the current temperature state of the object, and these errors can be found and solved in time, such as in healthcare. Therefore, highly accurate flexible temperature sensors are gathering numerous attentions in chronobiology study, medical application, predicting disease, monitoring postoperative recovery, etc (Kim, 1979; Busto et al., 1987; Michenfelder, 1991; Schwab, 1997; Mack, 2002; Marshall, 2006; Childs, 2008; Mrozek, 2012; Sheng et al., 2013; Wu et al., 2017; Oh et al., 2018). The main method of preparing highly accurate flexible temperature sensors is using sensing materials with high sensitivity to temperature. Commonly used high sensitivity sensing materials are high-crystallinity silicon or functional composites. In addition, the sensitivity of the flexible temperature sensor can be improved by introducing microstructures into the device to achieve high precision measurement. However, the fabrication process of this accurate flexible temperature sensor consisting of special materials or special structures is relatively complicated.

Wu group (Wu et al., 2017) demonstrated a highly accurate flexible temperature sensor with polysilicon thermistors on flexible PI to monitor brain’s temperature with high spatial resolution. The highly accurate flexible temperature sensor has a response time of 1.5 s and a sensitivity of −0.0031°C^−1^. The thermal hysteresis of this highly accurate temperature sensor in physiological temperature range of 30–45°C was less than 0.1°C. Using the passivation layer of silicon nitride, this highly accurate flexible temperature sensor exhibited drift of less than 0.3°C in water for 3 d. The performance of this highly accurate flexible temperature sensor showed a low noise level of 0.025 ± 0.03°C, and the expected transient increases in cortical temperature associated with cortical spreading depolarization. Highly accurate flexible temperature sensor developed in this research is desired to monitor brain’s temperature with high resolution and sensitivity.

Oh group (Oh et al., 2018) reported a highly accurate flexible temperature sensor with a bioinspired octopus-mimicking adhesive. The highly accurate flexible temperature sensor consists of a composite of CNTs, poly (N-isopropylacrylamide) (pNIPAM)-temperature sensitive hydrogel and poly (3,4-ethylenedioxythiophene) polystyrene sulfonate. The highly accurate flexible temperature sensor exhibited an ultrahigh thermal sensitivity of 2.6%°C^−1^ at 25–40°C, therefore a change of 0.5°C in skin’s temperature can be detected accurately. Simultaneously, the PDMS adhesive layer of octopus-mimicking rim structure coated with pNIPAM was fabricated *via* formation of a single mold through applying undercut phenomenon in photolithography. Without any skin irritation for a long time, the fabricated sensor showed reproducible and stable detection of skin’s temperature at repeated attachment/detachment cycles onto skin. This study demonstrated the application of highly accurate flexible temperature sensor in wearable devices for health-care and medical monitoring with a great potential.

### Stretchable Flexible Temperature Sensor

To ensure that no performance deterioration occurs due to body movements, the stretchable flexible temperature sensors are required, when stretchable flexible temperature sensors are applied to noncoplanar surfaces including robot’s body and human’s skin (Lee et al., 2014; Park et al., 2015; Tee, 2015; Chortos et al., 2016; Gao et al., 2016; Soekadar et al., 2016; Wehner et al., 2016; Gupta and Loh, 2017). The fabrication of a stretchable flexible temperature sensor with a high mechanical stability under strain is found to be a critical challenge since the change of sensitivity in stretchable flexible temperature sensor occured during stretching. For fabrication of stretchable flexible temperature sensor, stretchable electrical interconnections are challenges. Successful serpentine interconnections of a polymer-encapsulated thin metal film are investigated. To relieve strain which is externally applied onto the whole electronic device, the interconnections are effective tools. Lately, it is reported that the liquid metal interconnections embedded in a deformable polymer substrate can be widely utilized as highly conductive and stretchable electrical interconnections by facile fabrication.

**FIGURE 4:**
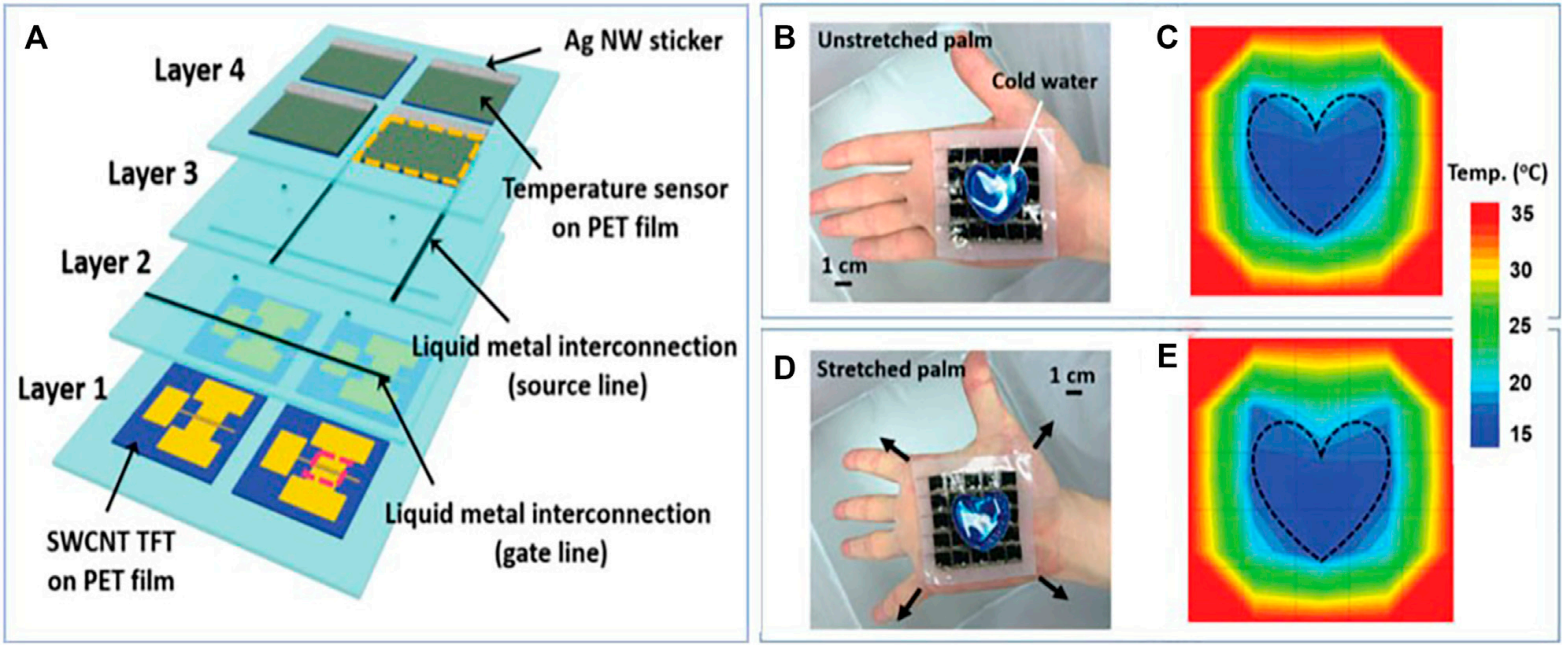
**(A)** The flat palm attached the encapsulated flexible temperature sensor array with a heart-shaped aluminum container, and the cold water (15 °C) is filled in the container. **(B)** Distribution of measured temperature of the flat palm by flexible temperature sensor array. **(C)** The unstretched palm attached the encapsulated flexible temperature sensor array with a heart-shaped aluminum container, and the cold water (15 °C) is filled in the container. **(D)** Distribution of measured temperature of the stretched palm by flexible temperature sensor array. **(E)** The stretched palm attached the encapsulated flexible temperature sensor array with a heart-shaped aluminum container, and the cold water (15 °C) is filled in the container. (Hong et al., 2016).

Hong group (Hong et al., 2016) reported the fabrication of a stretchable flexible temperature sensor array with liquid metal interconnections embedded in a deformable polymer substrate. In this study, fabricating a stretchable flexible temperature sensor with a stable performance at a strain up to 30% was available since the stretchable flexible temperature sensor’s sensitivity shows high stability during stretching. As shown in Figure 4A, the stretchable flexible temperature sensor consists of SWCNT TFT on PET film (layer 1), gate line (layer 2), source line (layer 3), temperature sensor on PET film and Ag NW sticker (layer4). As shown in Figures 4B,C,D,E, the corresponding mapping of the temperature distribution under stretched palm condition is consistent with the one of the temperature distribution under flat palm condition.

### Active-Matrix Flexible Temperature Sensor

According to storage devices including transistors or diodes, FRTCs can be classified as passive-matrix FRTCs and active-matrix FRTCs. Passive-matrix FRTCs have simple structure, usually electrode-substrate-sensing material sandwiched structure consisting of a sensing layer, an electrode and a substrate. Active-matrix FRTCs have complex structure which includes organic transistor, gate, thermistor, encapsulation, line and substrate. Active-matrix FRTCs are equipped with transistors or diodes for each unit (Kaltenbrunner et al., 2013). When the switch is turned on, the driving voltage of the specification can be transmitted to the unit. When the switch is turned off, the irrelevant signal can be cut off, so the crosstalk phenomenon can be greatly reduced. Among them, passive-matrix FRTCs are the most used devices in the construction of flexible temperature sensors based on conductive and also the most popular devices for practical applications, because of its simple structure, convenience to implement, and relatively low cost. As opposed to passive-matrix FRTCs, the active-matrix FRTCs allow individual and random access to each unit with high addressing speed and simultaneously maintaining a high density of device (Tsuyoshi et al., 2009; Sekitani, 2008; Zhang et al., 2015a; Ren et al., 2016).

**FIGURE 5:**
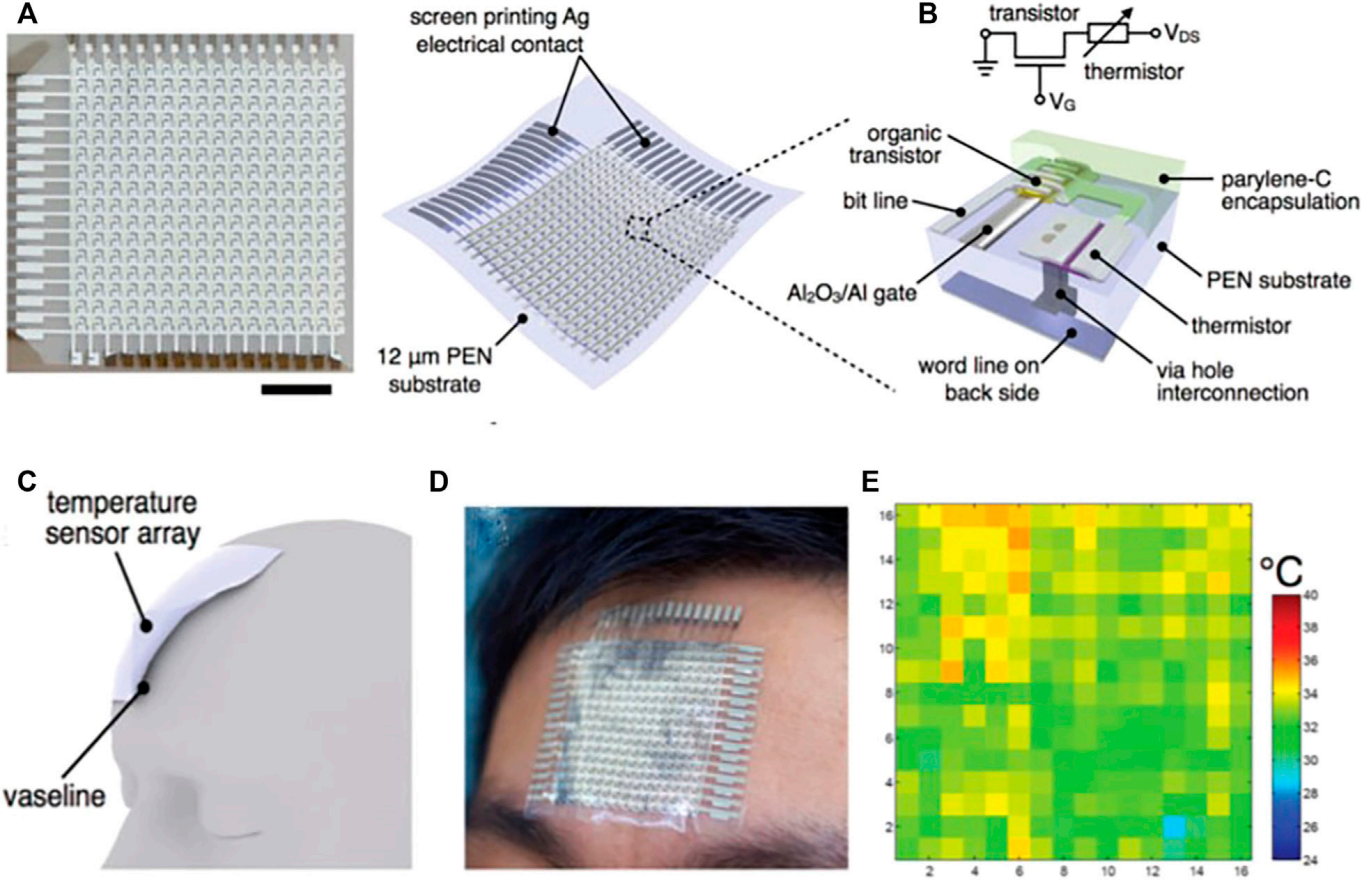
**(A)** Optical image and schematic drawing of flexible temperature sensor array (the scale bar is 10 mm). **(B)** Schematic of a flexible temperature sensor unit. **(C)** Schematic of flexible temperature sensor array attached to the forehead. **(D)** Optical image of the flexible temperature sensor array. **(E)** Corresponding mapping of distribution of the flexible temperature sensor array on the forehead (Ren et al., 2016).

Ren group (Ren et al., 2016) demonstrated an active-matrix FRTC array with organic field-effect transistor structure (Figures 5A,B). By utilizing a PEN substrate pentacene/silver NPs thermistor, and alumina dielectric, the sensor can be conformally attached to various objects and operated at blow 4 V, and a leakage current of about tens of pA is maintained. When changing the operating temperature from 20 to 100°C, this flexible temperature sensor array maintains more than 20 times the output-current change. As shown in Figures 5C–E, when the flexible temperature sensor is attached to a volunteer’s forehead, distribution of the measured temperature of the forehead could be obtained.

### Self-Powered Flexible Temperature Sensor

Self-powered materials enable the equipment to extend the service period through harvesting energy from body’s temperature and movement (Chen et al., 2017b; Cheng et al., 2018; Jayaweera et al., 2018; Liu et al., 2018). It is difficult to provide portable and durable power supply for flexible temperature sensors. At present, many advanced technologies, such as supercapacitors, solar cells, wireless antennas and mechanical energy harvesters, are found to be able to generate electricity and to transmit or store energy in elastic systems (Yang et al., 2013; Song et al., 2014; Chen et al., 2017c; Gong and Cheng, 2017). How to apply these technologies to flexible temperature sensors and realize energy self-supply is a huge challenge. Transparency of electronic skin tactile sensors can be achieved by using high transparency PDMS and other materials, which can ensure the absorption of energy by mechanical equipment driven by solar energy. Therefore, transparency design is also important. Flexible temperature sensors will also face new challenges, such as biocompatibility, biodegradability, neural interface control, high integration, miniaturization etc., which will become the research hotspots in future (Yang et al., 2009; Hochbaum and Yang, 2010; Chu and Majumdar, 2012; Pugliese et al., 2013; Hernandez et al., 2014; Nour et al., 2014; Yingkui et al., 2015; Ghosh et al., 2017; Maity et al., 2017; Nour et al., 2017; Yu et al., 2017; Gui et al., 2018; Karmakar et al., 2019). Flexible temperature sensor manufactured in large quantities is expected to enter all fields of human’s production and life, and truly serve human beings, which is the future direction of development.

**FIGURE 6:**
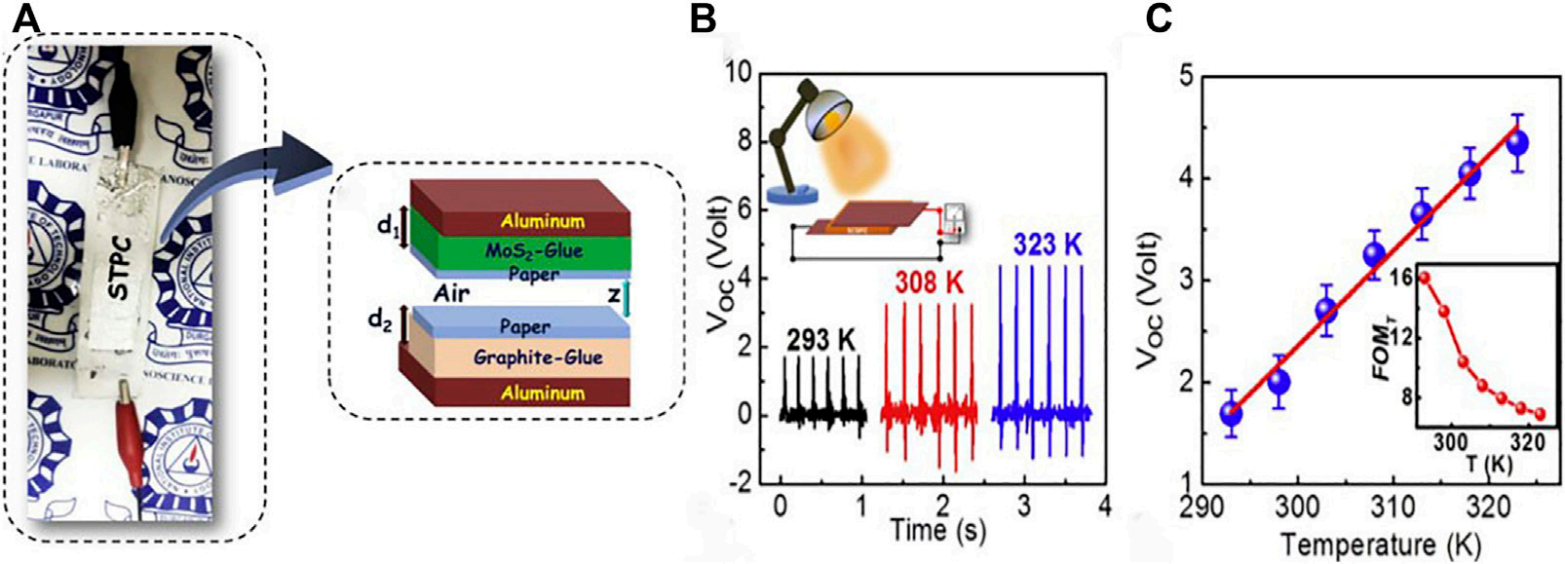
**(A)** Optical image and schematic drawing of STPC, **(B)** variation of average V_oc_ of STPC at 293, 308, and 232 K under a constant periodic pressure of 1 kPa, and the schematic of measurement technique is shown in the inset, **(C)** the linear fitted curve of V_oc_ *vs*. temperature at the temperature region of 293–323 K, and the relative sensitivity coefficient (S) of V_oc_ with temperature is shown in the inset (Karmakar et al., 2019).

Karmakar group (Karmakar et al., 2019) presented a new type of self-powered flexible temperature sensor consisting of self-charging and triboelectric driven flexible power cell. Commercially available materials are employed in the fabrication of this self-charging triboelectric power cell, such as non-conductive glue, bulk MoS_2_, normal sheet of paper and graphite powder (Figure 6A). The self-charging triboelectric power cell showed excellent output performance with open circuit voltage of ∼3.82 V at a periodic pressure of 1 kPa. The open circuit voltage (V_oc_) of the self-powered flexible temperature sensor is highly sensitive and has a liner response to temperature. As demonstrated in Figure 6B, the value of average open circuit voltage (V_oc_) increases with the increase of temperature during heating and cooling. It is observed from Figure 6C that the *d*V/*d*T of the self-powered flexible temperature sensor is 0.093 V K^−1^ in temperature range of 293–323 K.

### Self-Healing Flexible Temperature Sensor

Self-healing performances of the materials used in the wearable devices enable the extended usage periods if scratch or cut generates. It has high practical value in bionic robots, medical care and other fields. Through self-repairing, the service life of self-healing flexible temperature sensor can be prolonged. This function mainly introduces self-healing characteristics into elastic materials. Self-healing must occur at ambient conditions without any trigger or external stimulus. Herein, we discuss both intrinsic and extrinsic self-healing polymers. The intrinsic self-healing is based on molecular interactions (e.g., π-π stacking, metal-ligand coordination and hydrogen bonding), whereas the extrinsic self-healing polymers are dependent on the release of monomers and catalysts packed in vessels or capsules dispersed in an otherwise nonhealing polymer (Hart et al., 2014; Burattini et al., 2010). Although extrinsic self-healing materials are more efficient in recovering larger-scale damage compared with intrinsic materials, they are, however, less suitable for flexible thin devices because they are not easily fabricated, and their integration into fully functional applications-especially in health monitoring applications-is complicated. The intrinsic self-healing polymers are more advantageous due to their ability to reversibly heal themselves multiple times and functionalization of polymer with different self-healing groups (Woola, 2008; Yang and Urban, 2013; Abraham et al., 2013; Kristen Means1 et al., 2019). Although researchers have achieved self-repairing of flexible temperature sensors, their stability and sensitivity need to be improved.

### Self-Cleaning Flexible Temperature Sensor

The self-cleaning function of electronic skin tactile sensor is also of great significance. It has broad application prospects in robots, medical equipment and other fields. However, few results of the self-cleaning function of electronic skin tactile sensor have been reported. Abraham group (Abraham et al., 2013) showed a self-cleaning sensor composed of thermoresponsive double network nanocomposite (DNNC) membrane including poly (N-isopropylacrylamide) (PNIPAAm) and embedded polysiloxane NPs. When thermoresponsive PNIPAAm hydrogels is thermally cycled above and below its volume phase transition temperature (VPTT) of ∼33–35°C, this process will lead to the associated deswelling and reswelling respectively and self-cleaning of material’s surface. A. Kristen Means group (Kristen Means et al., 2019) demonstrated a self-cleaning biosensor consisting of 2-acrylamido-2-methylpropane sulfonic acid (AMPS) and N-isopropylacrylamide (NIPAAm) (ratios of AMPS: NIPAAm are 25: 75 and 0: 100) in the 1st and 2nd networks. Cellular attachment is inhibited by this reported membrane utilizing “self-cleaning” or “actively antifouling” mechanism through cyclic, continuous deswelling/reswelling in response to subcutaneous tissue’s normal temperature fluctuation (Kristen Means et al., 2019).

## Applications

**FIGURE 7:**
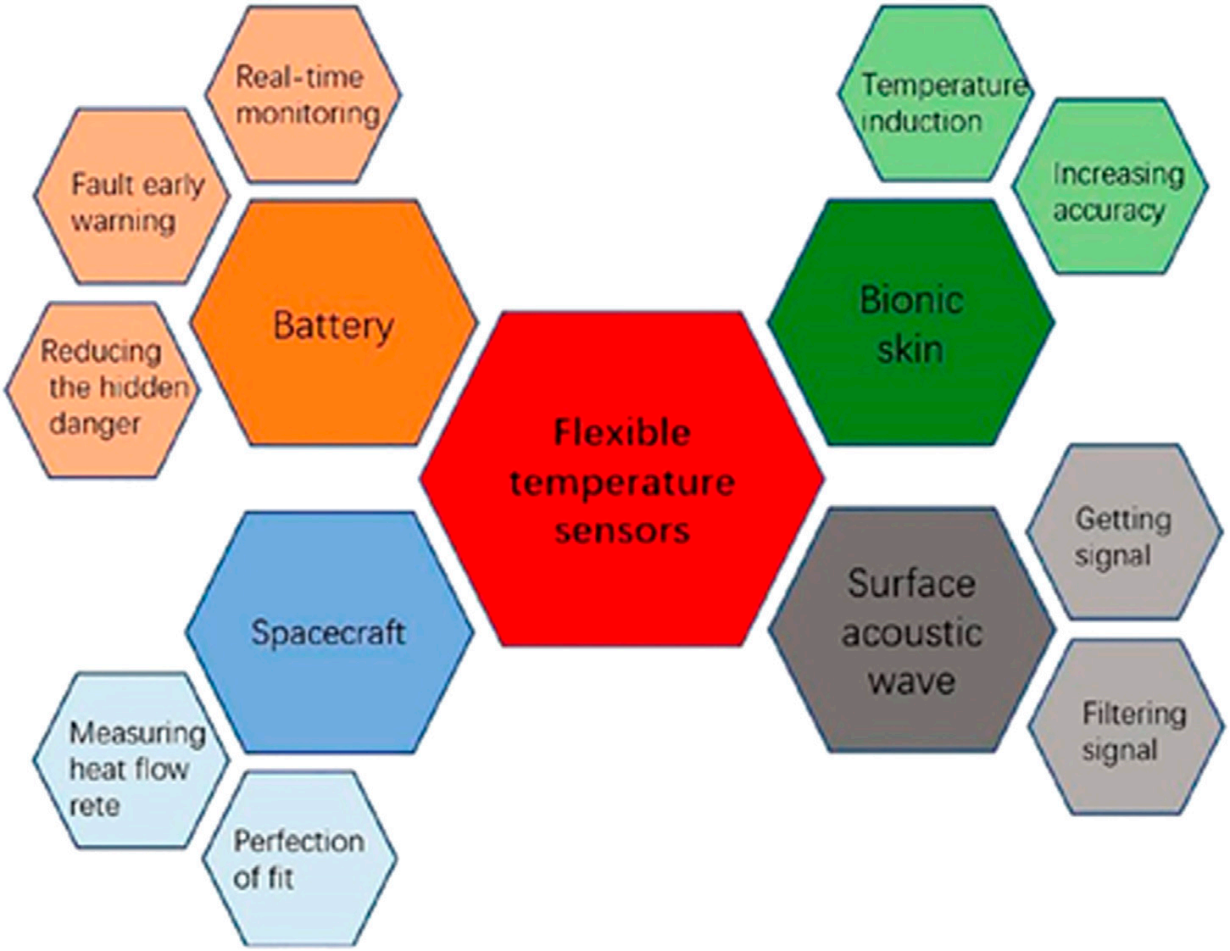
Applications of flexible temperature sensors.

Recent progresses in materials and fabrication allow the development of flexible temperature sensors with induction performances highly compatible with other functions, and allow the expansion of applications of flexible temperature sensors. Flexible temperature sensors are indispensable devices with stereotypical applications involving robots, medical health, military, intelligent manufacturing, aircraft safety and daily life (Zheng et al., 2019; Zhang et al., 2015b; Peter et al., 2015), as shown in Figure 7. The applications of flexible temperature sensors will lead to a reduction in cost and an increase in accuracy. Associated with bionic skin, surface acoustic wave, spacecraft and battery, a more comprehensive discussion of applications and importance is elaborated.

### Power System

In the power system, many major accidents are caused by overheating of electrical equipment (Jintae et al., 2014). Real-time monitoring of the temperature of electrical equipment can discover the hidden danger of overheating of electrical equipment, ensure timely maintenance, eliminate the hidden danger of failure as soon as possible, and greatly reduce the operation accidents of power supply system. Therefore, the safety of power supply area is improved.

#### Battery Temperature Monitoring

Battery is an emergency standby power supply for operation, control and communication of electrical equipments such as power plants and substations (Huda et al., 2013). High-temperature operation will accelerate the aging of batteries, and even have the risk of explosion. At the same time, there will be bulging, plate deformation and other faults. These battery failures will cause system failures such as operation, control, communication, and erroneous instructions, so it is very important to monitor the real-time temperature of the battery. At present, the temperature monitoring method of storage battery is mainly manual detection by infrared temperature detector, which is low in mechanization and high in cost, and can not realize on-line monitoring. Flexible temperature sensor can be attached to a surface of storage battery to realize the measurement of distributed temperature (Shin et al., 2013). This method is easy to conduct, and can realize fault monitoring and early warning, reducing the security risk of power system.

When the battery operates at high temperature for a long time, it is easy to accelerate the aging of the battery, and aggravate the corrosion of the plate and water loss (Atsushi et al., 2019). The flexible temperature sensor is used to paste on the surface of the battery, having the advantages of easy install, simple implement and easy integration with equipment. It can monitor the temperature of the battery in real time, warn the failure and reduce hidden dangers (Shih et al., 2010).

#### Applications of Capacitor Temperature Monitoring

Capacitor is an important component of reactive power compensation in power system. It plays an important role in improving power and reducing line loss (Lee et al., 2011b). However, due to the influence of various factors such as external working environment, current overload loss and over rated voltage operation, oil leakage will occur in long-term used capacitors (Pontus et al., 2011). Almost all capacitor faults such as belly bulging, fuse breaking and shell flashing, are accompanied by temperature rise (Lee et al., 2011a). Therefore, capacitor temperature monitoring can detect capacitor faults as early as possible and effectively avoid the power loss caused by capacitor faults. Traditional monitoring methods have some shortcomings, such as high cost, difficult installation and poor insulation (Mankay, 2010). Flexible temperature sensor can cover the surface of capacitor as a thin film topography, and measure the temperature accurately. It is easy to install and operate, and can realize real-time monitoring and early warning of faults effectively (Shin et al., 2013).

#### Cable Temperature Monitoring

In the power system, the cable is the main electrical equipment of power plants and substations, and its failure often causes large-scale power outages. Cable’s heat mostly occurs at the joint, because the current transmitted in the cable is larger, if the contact resistance is slightly increased, and the temperature will be high (Oprea et al., 2009). Therefore, it is necessary to ensure that all busbar joints are in good contact. Therefore, it is very important to monitor the joint temperature in real time to find out the fault of power equipment and to maintain power equipment in time. Flexible temperature sensors can be used as patches attached to the cable for accurate real-time monitoring of temperature, thereby reducing human consumption, improving the mechanization and efficiency of the power system, so as to more effectively prevent, monitor and repair cable faults (Jiang, 2017).

### Industrial Production

In industrial production, accurate measurement and control of temperature parameters are essential for output quality, production efficiency and safe operation. At present, the commonly used heat treatment and thermal processing are begun to use the flexible temperature sensor to replace the traditional temperature sensor, which has never realized the measurement and control of temperature in the production process or important production equipment.

#### Surface Acoustic Wave

One of the most concerning performances of surface acoustic wave is the fact that it can propagate along the dielectric’s surface. Based on transmitting or intercepting signals from the dielectric’s surface, signal processing functions such as filter and sensor can be realized. There is a positive correlation between temperature and frequency. We can obtain accurate signals from the dielectric’s surface attached flexible temperature sensors (Kun et al., 2014).

#### Spacecraft

With the development of aerospace technology, the shape and structure of aircraft become more and more complex. When measuring the surface’s heat flux, the distance between measured points cannot be smaller because of the size of sensor. Since the base material of thin film resistance temperature sensor is solid material such as glass and ceramics, the measuring end surface of sensor does not coincide well with the model surface, resulting in inaccurate measuring structure. If the sensor has a flexible base, it can solve the problem of measurement of heat flux on the surface of complex surface model to a certain extent. It can not only make the installation of the sensor more convenient, but also make the measurement of end surface coincide with the model surface better (Wang et al., 2015).

### Medical Device

The application of temperature sensor in medical electronics is also common. For example, a non-contact thermometer can measure the heat emitted from a remote infrared radiation heat source, a thermistor element temperature sensor for a blood analyzer can be employed for monitoring the temperature of chambers, diffuser lamps and oil-cooled motors in order to avoid overheating. With the development of technology, temperature sensor manufacturers can help designers to reduce the size of medical devices in four ways, including providing flexible packaging options, reducing the size of sensor integrated circuits, integrating multiple sensor functions and intelligent devices.

#### Bionic Skin

Bionic skin based on various sensory functions of human body is an important development direction in the field of bionics at present. Various bionic sensors can replace organism’s response to temperature, humidity and pressure through structure and function design. This has prompted the generation of various bionic sensors. One major bionic sensor is flexible temperature sensor. Application of flexible temperature sensors in bionic skin will provide a much-needed objective tool for temperature induction and help in increasing induction accuracy. A recent analysis of these studies highlighted the importance due to use of various flexible temperature sensors. The authors reported that the flexible temperature sensor array can realize tactile perception, and it provides a design scheme for bionic skin (Wu, 2015; Kumar et al., 2019).

#### Prosthetics

Prosthetics are essential tools for people with disabilities to gain normal abilities. The current prosthesis only has the function of moving, but it does not have the function of sensing. Flexible temperature sensors are small, highly integrated, and can adhere to surfaces of any shape. If a flexible temperature sensor is applied to an existing prosthesis, the disabled can not only move normally, but also sense the temperature of the object. The application of flexible temperature sensors greatly enhances the sensory experience of people with disabilities and reduces the risk of secondary injuries (Mallory et al., 2013b).

## Conclusion and Prospects

Flexible temperature sensors can be applied to robots, medical health, military, intelligent manufacturing, aircraft safety and daily life, and have broad application prospects. Flexible temperature sensors have many characteristics, such as high flexibility, high elasticity, high sensitivity, high resolution, and lightweight. Various sensing principles have been applied to the study of flexible temperature sensors, and have benefited from the emergence of new sensitive materials, new sensor structures and microstructures, as well as advanced technologies such as nano-fabrication and printing technology. Flexible temperature sensors have made breakthroughs in flexibility, sensitivity and multi-function. Most flexible temperature sensors utilizing an individual material only focused on the unitary state of mechanical stimuli or applied composites for multifunctional flexible temperature sensors. Increased manufacturing cost and complicated fabrication process will be obtained by this approach. Therefore, most flexible temperature sensors are still in the laboratory stage, and they are individual and isolate device, therefore, they are not really put into use to serve the human society. The existing flexible temperature array sensors still have difficulties in obtaining both high elasticity and high flexibility. Large-area flexible temperature sensors have poor scalability, are not easy to cut and splice, and have high sensitivity of electronic skin contact. The most important research directions of flexible temperature sensors are high sensitivity and multi-function, self-healing and self-cleaning, self-power supply and transparency (White et al., 2001; Rodriguez-Donate et al., 2011; Jie, 2012).
